# Promoting antihepatocellular carcinoma activity against human HepG2 cells via pyridine substituted palladium complexes: in vitro evaluation and QSAR studies

**DOI:** 10.55730/1300-0527.3536

**Published:** 2023-01-04

**Authors:** Öğünç MERAL, Fatih Mehmet EMEN, Emine KUTLU, Ruken Esra DEMİRDÖĞEN, Neslihan KAYA KINAYTÜRK, Görkem KISMALI, Şevkinaz DOĞAN

**Affiliations:** 1 Department of Biochemistry, Faculty of Veterinary Medicine, Ankara University, Ankara, Turkey; 2 Department of Chemistry, Faculty of Arts and Sciences, Burdur Mehmet Akif Ersoy University, Burdur, Turkey; 3 Department of Chemistry, Faculty of Science, Çankırı Karatekin University, Çankırı, Turkey; 4 Department of Nanoscience and Nanotechnology, Faculty of Arts and Sciences, Burdur Mehmet Akif Ersoy University, Burdur, Turkey; 5 Department of Nursing, Faculty of Health Sciences, Burdur Mehmet Akif Ersoy University, Burdur, Turkey

**Keywords:** Pyridine-palladium complexes, hepatocellular carcinoma, cell death, liver cancer

## Abstract

Bis(4-(4-nitrobenzyl)pyridine)dichloropalladium(II), [PdCl_2_L^1^_2_], bis(2-amino-5-bromopyridine)dichloropalladium(II), [PdCl_2_L^2^_2_], bis(2,4-dimethylpyridine)dichloropalladium(II), [PdCl_2_L^3^_2_], bis(3,4-dimethylpyridine)dichloropalladium(II), [PdCl_2_L^4^_2_] were prepared. The spectroscopic techniques (FT-IR and ^1^H-NMR, ^13^C-NMR) were used to characterize the compounds. Theoretical calculations were used to validate the experimental results. The LanL2DZ-based DFT/B3LYP method was used to define the most stable possible molecular structure for the complexes. Potential energy distribution analysis was performed to determine the theoretical vibration bands of the complexes. Molecular electrostatic potential maps, boundary molecular orbitals and Mulliken charge distribution were used to determine the active sites of the molecules. The interaction mechanisms between the complexes and liver cancer protein were investigated via molecular docking. The study on the antiproliferative effects of these complexes on hepatocellular carcinoma cells (HepG2) showed that they are potent candidates for use against this liver cancer cell line.

## 1. Introduction

Cancer, which is a complex disease characterized by uncontrolled cell proliferation, is the second largest cause of mortality in the world [1, 2]. Among other cancer types hepatocellular carcinoma (HCC), which is the most common type of primary liver cancer in adults, was the third most common cause of cancer deaths in 2020 in the world [3]. The highest incidence and the lowest survival rate after treatment for HCC was observed in Asia and sub-Saharan Africa where hepatitis B infection is endemic [4]. It is foreseen that new cancer cases will increase significantly in the next decades [4–7]. There are many types of cancer treatment options which are usually very complex and they are developing gradually. Among them are chemotherapy, surgery, and radiation therapy. Chemotherapy entails use of special drugs to kill cancer cells and has been recently supported by new methods of treatment such as immunotherapy to give successful results. However, chemotherapeutics have many negative side effects which compromises effectiveness of this treatment method. Therefore, recently increasing importance and efforts are devoted to development of more effective chemotherapeutic drugs with fewer side effects [8,9].

Metal-based compounds have attracted much attention since *cis*-platin was discovered by Barnett Rosenberg in 1960 and they have been used widely in treatment of various cancer types, especially of head and neck, ovarian and colorectal cancers. Today, *cis*-platin and its derivatives (i.e. carboplatin and oxaliplatin) are still extensively used in cancer treatment [10]. However, these compounds have major disadvantages such as the limitation of their efficacy to some cancer types as well as their extensive side effects [11]. Hence, recently new studies on other metal complexes have increased. Among these metal complexes, cost effective palladium complexes have attracted much attention as they form compounds similar to platinum [12]. Studies revealed that palladium (II) complexes have considerable anticancer activity on various cancer cell lines [13]. The many different types of Pd (II) complexes (i.e. monomeric, dimeric, tetrameric, cyclo-palladated, palladacyclic, and heterobimetallic) were reported to have cytotoxic activity and the pyridine, pyrazole, quinoline and, 1,10-phenanthroline derivates of Pd (II) have great antiproliferative activity and all of them are promising anticancer agents as these complexes are more soluble than platinum complexes [14]. Despite the large number of reports on pyridine derivative complexes (i.e. chloropyridine, bromopyridine [15], methylpyridine [16], (p-tolyl) pyridine [17], (2,4 dinitrobenzyl) and ((2,4,6 trinitrobenzyl) pyridine) [18] only a very few of them are related to their anticancer effect. Studies showed that these pyridine derivatives can be exploited as anticancer drugs [19] with the effective mechanism of action of Pd complexes that proceeds over inhibition of cell proliferation of cancer cells via DNA binding [14]. Tabrizi et al. reported that the palladium complexes (II) with 2,2,-bipyridine (bpy) ligands have remarkable cytotoxic activity against the colorectal adenocarcinoma (HT-29), the breast (MCF-7), and the human squamous cervical adenocarcinoma (HeLa) cancer cell lines which was better than the effect of cisplatin [20]. In a study by Kuduk-Jaworska et al., palladium(II) complexes with 2,6-dimethyl-4-nitropyridine complexes were observed to have strong antiproliferative effect against adenocarcinoma of the rectum (SW707), breast cancer (T47D), bladder cancer (HCV) and nonsmall cell lung carcinoma (A549) cancer cell lines [21]. Franich et al. investigated the antiproliferative activity of palladium(II) with 4,4,-bipyridine ligands on murine lung cancer (LLC1 cells) and they reported that they had similar cytotoxic effect to cisplatin [22].

In this study, bis(4-(4-nitrobenzyl)pyridine)dichloropalladium(II), [PdCl_2_L^1^_2_], bis(2-amino-5-bromopyridine) dichloropalladium(II), [PdCl_2_L^2^_2_], bis(2,4-dimethylpyridine)dichloropalladium(II), [PdCl_2_L^3^_2_], bis(3,4-dimethylpyridine) dichloropalladium(II), [PdCl_2_L^4^_2_] were synthesized and characterized via spectroscopic techniques (FT-IR and ^1^H-NMR, ^13^C-NMR). The antiproliferative effects of these complexes on hepatocellular carcinoma cells (HepG2) were investigated and the interaction mechanisms of liver cancer protein and complexes were investigated by molecular docking studies.

## 2. Experimental

### 2.1. Materials

All chemicals and solvents used were of high purity and were used without further purification. PdCl_2_ was supplied from Sigma Aldrich and 4-(4-nitrobenzyl)pyridine, 2-amino-5-bromopyridine, 2,4-dimethylpyridine, 3,4-dimethylpyridine and ethanol were supplied from Merck.

### 2.2. Synthesis of the complexes

The complexes were synthesized by reacting PdCl_2_ with the pyridine derivatives in ethanol (Scheme). A solution of PdCl_2_ (1.2 mol) was prepared in 50 mL of distilled water. The ethanolic solutions of the ligands (30 mL) were prepared by dissolving 2.4 mol of the ligands (L^1^*:*4-(4-nitrobenzyl)pyridine, L^2^*:*2-amino-5-bromopyridine, L^3^*:*2,4-dimethylpyridine, L^4^*:*3,4-dimethylpyridine). These solutions were added to the palladium solution under constant stirring and reflux for 2 h. Then thus obtained precipitates were filtered and dried.

#### Bis(4-(4-nitrobenzyl)pyridine)dichloropalladium(II), [PdCl_2_L^1^_2_]

FT-IR (KBr, ν_max_, cm^−1^): 3080 ν(Ar-C–H), 2925–2854 ν(Al-C–H), 1614–1537 ν(Ar-C = C, C = N), 1350 ν(N–O), 1110–1016 δ(C–H in-plane), 880–518 δ(C–C in-plane), 470 ν(Pd–N). ^1^H-NMR (d6-DMSO, ppm): 8.64–7.42 (16 H, Ar-H), 4.24 (4 H, Al-H). ^13^C-NMR (d6-DMSO, ppm): 152.5 (4 C, o-Ar-C), 149.4 (p-Ar-C),146.0 (C-NO_2_), 146.2 (Ar-C), 130.1 (Ar-C), 125.3 (m-Ar-C), 123.6 (Ar-C), 40.0 (CH_2_). TOF-MS (ESI^+^) for C_24_H_20_Cl_2_N_4_O_4_Pd (Acetonitrile), [M+7 H]^+^, *m/z*: calcd. 612.8246 found 612.077.

#### Bis(2-amino-5-bromopyridine)dichloropalladium(II), [PdCl_2_L^2^_2_]

FT-IR (KBr, ν_max_, cm^−1^): 3427–3180 ν(N–H), 3076 ν(Ar-C–H), 2985–2839 ν(Al-C–H), 1622–1402 ν(Ar-C = C, C = N), 1265–1145 δ(C–H in-plane), 889 ν(C–Br), 823–503 δ(C–C in-plane), 476 ν(Pd–N). ^1^H-NMR (d6-DMSO, ppm): 8.76–6.14 ( 6 H, Ar-H), 7.64 (4 H, N-H). ^13^C-NMR (d6-DMSO, ppm): 158.1 (C -NH_2_), 149.3 (o-Ar-C), 140.5 (p-Ar-C), 112.3 (m-Ar-C), 103.5 (C-Br).

#### Bis(2,4-dimethylpyridine)dichloropalladium(II), [PdCl_2_L^3^_2_]

FT-IR (KBr,ν_max_, cm^−1^): 3030 ν(Ar-C–H), 2950–2886 ν(Al-C–H), 1600–1473 ν(Ar-C = C, C = N),1251–1043 δ(C–H in-plane), 885–657 δ(C–C in-plane), 503 ν(Pd–N). ^1^H-NMR (d6-DMSO, ppm): 8.40–7.65 (6H, Ar-H), 3.31-2.26 (12H, Al-H). ^13^C-NMR (d6-DMSO, ppm): 150.2 (o-Ar-C), 140.6 (p-Ar-C), 134.9 (m-Ar-C), 17.9 (-CH_3_). TOF-MS (ESI^+^) for C_14_H_18_Cl_2_N_4_O_4_Pd (Dichloromethane), [M+6 H]^+^, *m/z*: calcd. 397.6774 found 398.0350.

#### Bis(3,4-dimethylpyridine)dichloropalladium(II), [PdCl_2_L^4^_2_]

FT-IR (KBr, ν_max_, cm^−1^): 3035 ν(Ar-C–H), 2990–2806 ν(Al-C–H), 1612–1415 ν(Ar-C = C, C = N),1250–1089 δ(C–H in-plane), 856–524 δ(C–C in-plane), 433 ν(Pd–N).^1^H-NMR (d6-DMSO, ppm): 8.45–7.30 ( 6H, Ar-H), 3.31–2.24 (12H, Al-H). ^13^C-NMR (d6-DMSO, ppm): 151.5 (o-Ar-C), 149.9 (p-Ar-C), 134.2 (m-Ar-C), 18.5 (-CH_3_).

### 2.3. Instrumentation

FTIR spectra (4000 to 400 cm^−1^) were recorded using the Perkin Elmer Frontier FTIR spectrometer. Samples were taken in KBr pellets. NMR spectra were recorded using the Bruker Ultrashield Plus Biospin Avance III 400 MHz NanoBay FT-NMR instrument. d6-DMSO was used as solvent. Full mass analysis was performed by scanning in the positive (ES+) 50 – 1000 Da range via High Resolution Mass Spectrometry using Waters SYNAPT G1 MS system.

### 2.4. Cell culture studies

The HepG2 cell line was obtained from ATCC (Wesel, Germany, Cat. HB-8065) and maintained in RPMI-1640 medium with L-Glutamine (584 mg L^−1^) (Irvine Scientific, Santa Ana, CA, USA) containing 10% (w/v) FBS (Irvine Scientific, Santa Ana, CA, USA) and gentamicin sulfate solution (50 μg mL^−1^) (Irvine Scientific, Santa Ana, CA, USA). Seventy-five square centimeters cell culture flasks (BD Falcon, Rockville, MD, USA) were used to grow the cells and the process was carried out in a humidified (5% CO2) incubator at 37 °C. The culture medium was changed every two days. Cells were subcultured with trypsin-EDTA solution (1:4, v/v) (Irvine Scientific, Santa Ana, California, USA).

### 2.5. Cell proliferation/viability assay

One hundred microliters of culture medium, 96-well flat-bottom cell culture plates (Greiner Bio One, Frickenhausen, Germany) at a density of 1 × 105 cells/well were used for seeding HepG2 cells. After 24 h of incubation, both unbound and dead cells were removed by washing twice with buffer solution (PBS, Irvine Scientific, Santa Ana, California, USA) prior to all assays. The cytotoxic effect of the complexes was determined by measuring mitochondrial dehydrogenase activity in HepG2 cells using methyl thiazolyl tetrazolium (MTT) as substrate. Different concentrations of the complexes were dissolved in RPMI-1640 medium (3.12, 6.25, 12.5, 25, 50, 100, and 200 μM) and were added to the cells which were then treated for 24 h. After incubation, cells were washed with fresh medium. One hundred microliters of MTT (5 mg mL^−1^) solution was added to all control and experimental cell groups. After 4 h of incubation, sodium dodecyl sulfate solution (10% (w/v), 100 μL) was added to each well to dissolve the formazan salt. The amount of formazan salts was quantified by measuring the absorbance value sat 570 nm in a microtiter plate reader (Sunrise, Tecan GmbH, Austria). IC50 values were calculated according to the MTT Assay results. Cell viability analysis was performed via one-way ANOVA followed by unpaired Student t-test. The IC50 values were calculated according to nonlinear quadratic model. p values <0.05 were considered to be statistically significant. Each experiment was performed in triplicates.

### 2.6. Evaluation of cell injury

Cell damage was assessed by measuring the release of lactate dehydrogenase (LDH) from the cells to the bath medium consisting of LDH. Cells were taken into 96-well plates and were incubated for 24 h after adding the complexes at different concentrations (3.12, 6.25, 12.5, 25, 50, 100, and 200 μmol L^−1^). LDH concentration in the Supernatant was determined by using Clinical Chemistry Analyzer (ERBA XL 600, Meinheim, Germany) and a commercial colorimetric assay kit (TML Medical, Ankara, Turkey). Total LDH release, which caused HepG2 cell death, was determined. Each experiment was performed in triplicates.

### 2.7. Computational details

Theoretical calculations were performed using Gaussian 09 software [23]. Quantum chemical calculations were performed using the B3LYP functional and LanL2DZ basis set and the density functional theory (DFT) method. B3LYP vibrational wave-numbers were found to be higher than experimental wave-numbers. Therefore, the scaling factor was used for the wave numbers and the mismatch effects were not taken into consideration. The wave-numbers calculated by B3LYP/Lanl2DZ bases set were scaled in the infrared spectra by 0.96, 1, 0.98, and 0.85 in the ranges [4000–2001] cm^−1^, [2000–1407] cm^−1^, [1406–341] cm^−1^ and [340–0] cm^−1^, respectively [24]. In general, the scaled wave-numbers were calculated and were found to be in good agreement with the experimental ones. The geometry was optimized after performing the frequency calculations by using the same basis set. The results were visualized by using Gauss View software [25]. Total energy distribution was calculated using VEDA software [26]. The 3D crystal structure of the targeted protein (PDB ID: 2OH4) was obtained using the RSBC PDB format. The optimized structures of the complexes were determined using the DFT/B3LYP/LanL2DZ basis set. Molecular docking studies were performed using Hex (version 8.0.0) software [27]. In molecular docking studies FFT mode: 3D fast lifetime; distance range: 40; twist range: 360; correlation type: shape + electro + DARS; grid size: 0.6; receptor spacing: 180 and ligand spacing: 180 parameters were used. PyMOL molecular graphics software was used to visualize the data obtained from the HEX 8.00 software [28].

## 3. Results and discussion

### 3.1. Synthesis of the complexes

The complexes were synthesized according to the method given elsewhere [19]. The pyridine derivatives were reacted with the palladium chloride solution prepared in ethanol by refluxing. The formation of these complexes is pH-dependent and there were obtained in the pH range 2–3. Therefore, the pH of the media was adjusted to 2–3 via ammonia and the brown colored complexes were separated after precipitating. After washing with ethanol a few times they were dried in the oven at 70 °C. In our previous work [19] the complexes were not further purified since they could have been precipitated in the pure form.

### 3.2. Structural analysis of the complexes

The FT-IR spectra of the complexes were obtained in the range 4000–400 cm^−1^. The experimental FT-IR data of the complexes are presented in Table 1 and the vibrational bands conferred to them are presented in Table S1 (in supporting information) in detail.

For all the complexes, while the vibrational band observed at 3030–3080 cm^−1^ indicates the Ar-H streching in the pyridine (Py) ring, the weak bands observed at 2990–2806 cm^−1^ indicates the C-H stretching vibrations of the aliphatic C-H groups. The intense bands observed in the range 1622–1402 cm^−1^ belong to the aromatic C=C and C=N stretching vibrations in the pyridine (Py) ring. The bands observed in the range 1265–1016 cm^−1^ show the in-plane C-H bending vibrations. The bands observed in the range 880–503 cm^−1^ indicate the C-C bending vibrations (in-plane). The vibrational band at 503–433 cm^−1^ indicates the Pd-N stretching vibrations. Beside the common stretching vibrations of the synthesized complexes the intense peaks observed with the [PdCl_2_L^1^_2_] complex at 1350 cm^−1^ indicate the N-O stretching vibrations of the nitro group. For the [PdCl_2_L^2^_2_] complex, the intense bands observed in the range 3427–3180 cm^−1^ indicate the N-H stretching vibrations of the NH_2_ group bound to the Py ring at the ortho position. The weak bands observed at 889 cm^−1^ indicate the C-Br stretching vibrations. These experimental findings were supported with the theoretical calculations. The theoretical studies were performed via the LanL2DZ based DFT/B3LYP method. The results of the theoretical calculations indicate that the N-H and C-H stretching vibrations should appear in the ranges 3570–3357 and 3135–3092 cm^−1^, respectively. The vibrational bands for C-Br, Pd-Cl and Pd-N, which are characteristic for this complex, were observed to lie in the fingerprint region. The theoretical potential energy distribution (%PED) showed that the vibrational bands of C=C, C=N and C-C overlapped. For the [PdCl_2_L^3^_2_] complex, the calculation showed that the C-H stretching vibrations were to appear in the range 3363–2965 cm^−1^. These bands were observed as medium and weak intensity bands in the FTIR spectra. The vibrational bands conferred to the in-plane C-C and C-H bending vibrations are presented in Table S1 in detail. For the [PdCl_2_L^4^_2_] complex, the C-H stretching vibrations of [PdCl_2_L^4^_2_]—a structural isomer of the [PdCl_2_L^3^_2_] complex—were calculated to be in the range 3141–2918 cm^−1^. The results of the calculations showed that while the in-plane C-H stretching bands would appear in the range 1520–1448 cm^−1^, the C-H bending vibrations were to be observed in the range 1073–852 cm^−1^. The Pd-Cl stretching vibration, which is characteristic for this complex, was calculated to appear at 415 and 409 cm^−1^. The obtained experimental and theoretical results are compatible with each other.

### 3.3. Molecular geometry studies

The optimized bond lengths and bond angles of the complexes are given in Table S2. Mainly five different bond lengths were observed in common for the C-C, C-H, C-N, N-Pd, and Pd-Cl bonds for these complexes. The length of the Pd-Cl and Pd-N bonds for all of the four complexes were calculated to be the same and are 2.27 and 2.05 Å, respectively. When all the bond lengths were examined, the longest bond length was determined to be the one between the palladium and the chlorine atoms. For the [PdCl_2_L^1^_2_] complex, the shortest bond length, which was calculated to be 0.96 Å, was observed to belong to the O-H bond. In other complexes, the length of the bond between the carbon and hydrogen atoms was calculated to be 1.07 Å. It can be seen in Table S2, the bonding angles between the Cl-Pd-Cl, Cl-Pd-N and N-Pd-N atoms are 90°. These bonding angles are the smallest for all the four complexes.

### 3.4. NMR studies

The ^1^H-NMR and ^13^C-NMR spectra of the complexes obtained in DMSO, which was used as the solvent, are presented in Table 2. The results of the theoretical calculations are presented in Table S3 in detail.

In the ^1^H-NMR spectrum of the [PdCl_2_L^1^_2_] complex, the multiple peaks observed in the range 8.64–7.42 ppm can be attributed to the Ar-H (16 H) peaks in the Py ring. The peaks observed at 4.24 ppm indicate the Al-H protons (4 H). The theoretical ^1^H-NMR values for this complex were calculated to be in the range 9.48–4.07 ppm.

In the ^13^C-NMR spectrum, the peak observed at 152.5 ppm shows the two C atoms of the complex in the o-position. The peak observed at 149.4 ppm shows the two C atoms in the p-position and the peak at 146.0 ppm shows the two C atoms to which the nitro group in the phenyl ring is bound. The other peak observed at 146.2 ppm indicates the two C atoms via which the phenyl ring is bound to the -CH_2_ bridge. The peak observed at 130.1 ppm indicates the four C atoms of the aromatic carbon in the phenyl ring. The peak at 125.3 ppm indicates the four C atoms in the metaposition. The peak at 123.6 ppm shows the four C atoms in the other aromatic carbon in the phenyl ring. The peak at 40.0 ppm indicates the two C atoms in the aliphatic groups of the complex. When these results are compared with the theoretical data it was seen that these values were distributed in the range 163–50 ppm and the chemical shifts observed in the ^13^C-NMR spectra of the structures were >100 ppm as expected according to the calculations.

The multiple peaks observed in the range 8.76–6.14 ppm in the ^1^H-NMR spectrum of the [PdCl_2_L^2^_2_] complex show the Ar-H (6 H) peaks in the Py ring. The singlet peak observed at 7.64 ppm shows the protons (4 H) in the amine group. In the ^13^C-NMR spectrum of the complex, the peak belonging to the two C atoms in the amine group at the o-position of the ring was observed at 158.1 ppm. The peak for the two C atoms in the other o-position was observed at 149.3 ppm. The peak for the two C atoms in the p-position was observed at 140.5 ppm. The peak for the two C atoms in the m-position was observed at 112.3 ppm. The peak observed at 103.5 ppm shows the two C atoms in the halogen groups of the complex.

The multiple peaks observed in the range 8.40–7.65 ppm in the ^1^H-NMR spectrum of the [PdCl_2_L^3^_2_] belong to the Ar-H (6 H) in the Py ring. These values were calculated to be in the range 9.76–7.99 ppm theoretically. The peaks observed in the range 3.31–2.26 ppm indicate the protons (12 H) in the methyl group. Theoretically they were found to be in the range 5.96–1.37 ppm.

In the ^13^C-NMR spectrum, the peak observed at 150.2 ppm shows the four C atoms in the o-position. The peak of the two C atoms in the para position was observed at 140.6 ppm. The peak observed at 134.9 ppm indicates the four C atoms in the m-position. The peak at 17.9 ppm shows the four C atoms in the methyl groups. Theoretically they were calculated to be in the 174.51–26.77 ppm range.

In the ^1^H-NMR spectrum of the [PdCl_2_L^4^_2_] complex, the multiple peaks observed in the range 8.45–7.30 ppm indicate the Ar-H (6 H) peaks in the Py ring. The peaks observed in the range 3.31–2.24 ppm indicate the protons (12 H) in the methyl group.

In the ^13^C-NMR spectrum, the peak observed at 151.5 ppm shows the four C atoms in the o-position. The peak at 149.9 ppm shows the two C atoms in the p-position. The peak at 134.2 ppm indicates the four C atoms in the m-position. The peak at 18.5 ppm shows the four C atoms in the methyl group.

### 3.5. Investigation of the MEP surface and Mulliken charge distribution

Molecular electrostatic potential surface maps (MEP), which allow us to observe the variable charge region, show the charge distributions of molecules in three dimensions.

**Figure 1:**
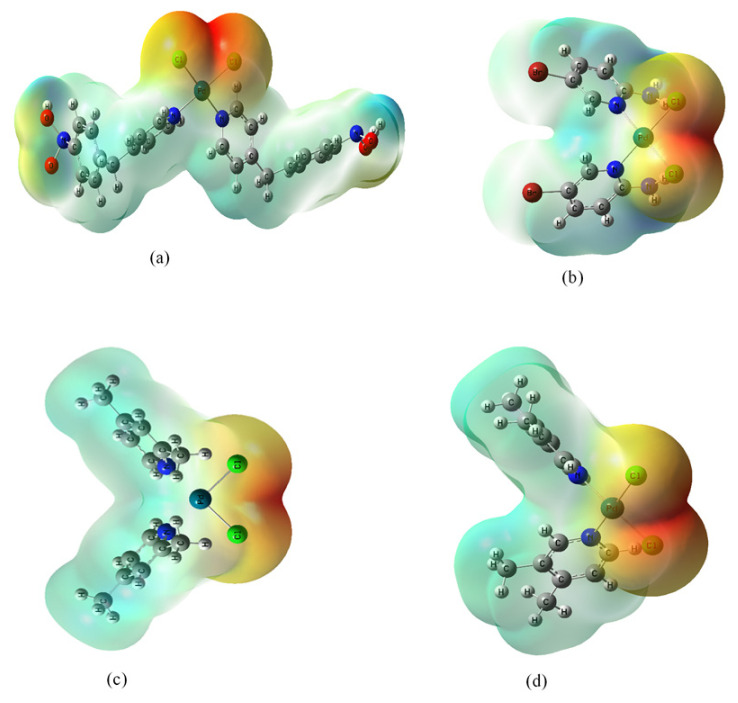
The colored coded picture of the electrostatic potential surface of the complexes (a) PdCl_2_L^1^_2_, (b) PdCl_2_L^2^_2_, (c) PdCl_2_L^3^_2_, (d) PdCl_2_L^4^_2_.

Mulliken atomic charge distributions of the complexes are given in Table 3. Figure 1 shows that the MEP surfaces of the complexes range from the darkest red to the darkest blue. Blue, red, and green colors indicate nucleophilicity, electrophilicity, and hydrogen bond interactions (regions of neutral or zero electrostatic potential), respectively [29,30].

In the MEP map, it is observed that the negative potential regions in the molecule are concentrated on oxygen and chlorine atoms. Complexes with such active sites generally show good biological activity. The Mulliken population analysis was performed in detail and the relevant results are given in Figure 1.

### 3.6. Investigation of the frontier molecular orbitals (FMOs) and the chemical reactivity

**Figure 2:**
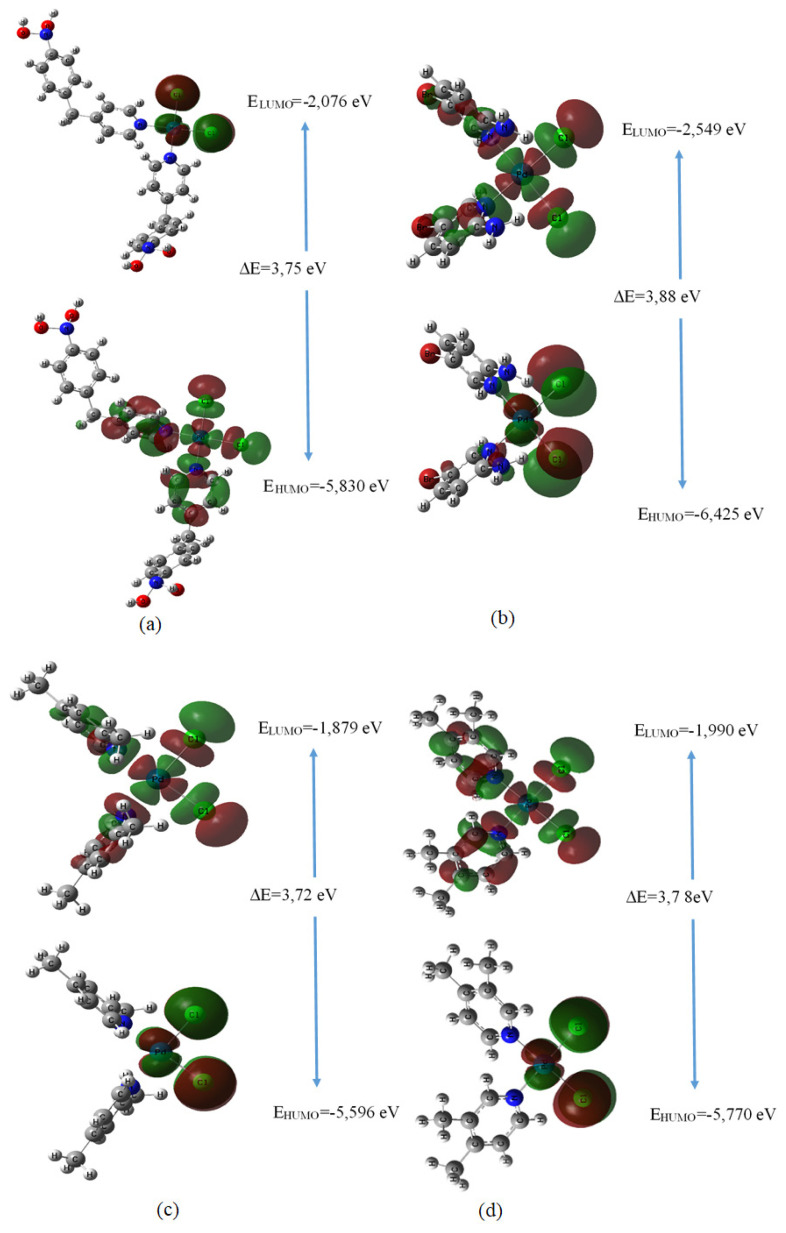
The HOMO and the LUMO surface images of the complexes (a) [PdCl_2_L^1^_2_], (b) [PdCl_2_L^2^_2_], (c) [PdCl_2_L^3^_2_], (d) [PdCl_2_L^4^_2_].

The difference between HOMO and LUMO energies determines the chemical reactivity and kinetic stability of a molecule. Moreover, with the calculated HOMO-LUMO values, the polarization, electronegativity, hardness, and reactivity of the energy gap molecule can be determined. The surface images are presented in Figure 2. The chemical reactivity indices are given in Table 4. A smaller HOMO-LUMO energy deficiency can indicate greater biological activity [31,32]. For the [PdCl_2_L^1^_2_], [PdCl_2_L^2^_2_], [PdCl_2_L^3^_2_], [PdCl_2_L^4^_2_] complexes while the HOMO energies were calculated to be −5.83, −6.43, −5.60 and −5.77 eV; the LUMO energies were calculated to be −2.08, −2.55, −1.88, and −1.99 eV, respectively. Also, the energy gap between the HOMO and LUMO orbitals were calculated to be 3.75, 3.88, 3.72 and 3.78, respectively.

### 3.7. Investigation of the cytotoxicity

**Figure 3:**
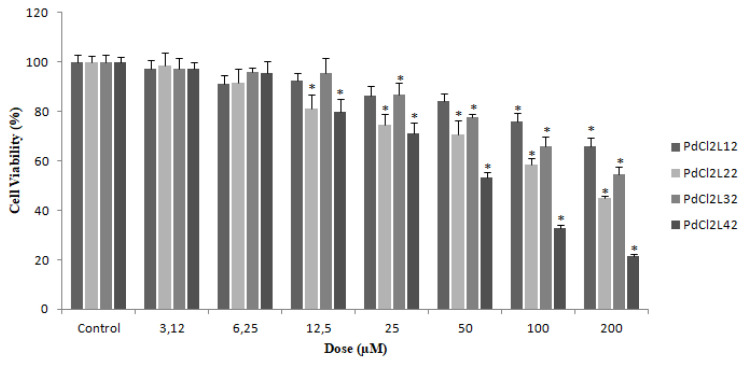
Viability of HepG2 cells treated with different doses of the complexes for 24 h. (*p < 0.05 indicates the significance of difference as compared with that of the control).

The effect of the complexes on cell proliferation of the HepG2 cells was investigated via MTT Assay. Half maximal inhibitory concentrations (IC50) of complexes were determined according to the MTT Assay results. IC50 values of [PdCl_2_L^1^_2_], [PdCl_2_L^2^_2_], [PdCl_2_L^3^_2_] and [PdCl_2_L^4^_2_] complexes were found to be 498.69 μmol L^−1^, 157.21 μmol L^−1^, 216.5 μmol L^−1^ and 61.04 μmol L^−1^, respectively. The change in cell viability was observed to be concentration dependent and also was affected from the structure of the complexes. HepG2 cells were interacted with complex solutions of different concentrations (3.12, 6.25, 12.5, 25, 50, 100, and 200 μmol L^−1^). The proliferation of the HepG2 cell line is suppressed depending on the added complex dose. Moreover, as can be seen in Figure 3, the data obtained from the cell viability study indicated that bis(dichlorobis(3,4-dimethylpyridine) palladium(II), [PdCl_2_L^4^_2_] was the most cytotoxic complex and bis(4- (4-nitrobenzyl)pyridine)dichloropalladium(II), [PdCl_2_L^1^_2_] was the least cytotoxic. In recent years, the cytotoxic activities of many types of complexes against various cancer cell lines have been studied. Among them palladium complexes (II) with 2,2,-bipyridine (bpy) ligands were found to be cytotoxic against colorectal adenocarcinoma and breast cancer cell lines [20]. In addition cytotoxic activity of palladium(II) complexes with 2,6-dimethyl-4-nitropyridine complexes and palladium(II) with 4,4,-bipyridine ligands were observed against several cancer cell lines in a dose-dependent manner [21, 22]. In this research, each complex suppressed the proliferation of HepG2 cell line in a dose-dependent manner with parallel to similar studies in the literature.

### 3.8. Investigation of the effect of complexes on cell injury

**Figure 4:**
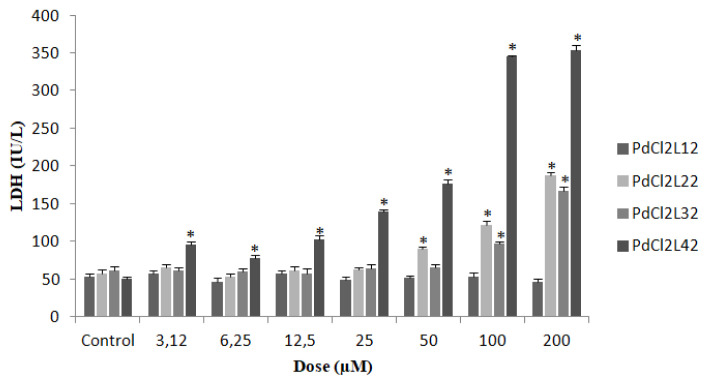
Cell culture supernatant lactate dehydrogenase (LDH) levels after 24-h incubation period. (*p < 0.05 indicates the significance of difference as compared with that of the control).

Cell injury was investigated via LDH assay conducted in culture media since LDH is a stable cytosolic enzyme in normal cells and when membrane damage occurs it leaks into the extracellular fluid. According to the cell viability assay bis(dichlorobis(3,4-dimethylpyridine) palladium(II), [PdCl_2_L^4^_2_] was the most cytotoxic complex and this complex increased LDH release—a cell damage marker—remarkably. The LDH release caused by bis(2-amino-5-bromopyridine) dichloropalladium(II), [PdCl_2_L^2^_2_] complex was the second highest among all the complexes studied. The LDH concentration (IU/mL) in the supernatant is given in Figure 4.

### 3.9. Molecular docking studies

The molecular docking technique describes the interactions between drug and enzyme and is therefore used in drug design studies [33]. Pd-based drugs are used in treatment of various cancers [34,35]. In this study, the interaction mechanism between the synthesized palladium complexes and the 2OH4 encoded HepG2 protein was investigated. The 3D crystal structure of the protein (PDB ID: 2OH4) targeted for HepG2 was observed using the RSBC PDB format.

**Figure 5:**
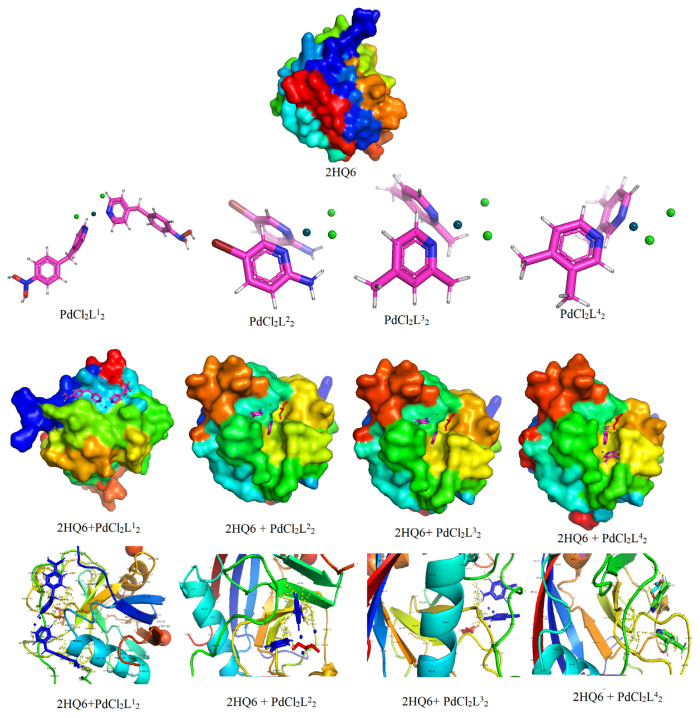
Docking diagram of the 2HQ6 target protein and the complex-protein interactions

The DFT/B3LYP/LanL2DZ basis set was used to optimize the structures of the synthesized complexes. The interaction energies between the complexes and the protein arise from the hydrogen bonds and van der Waals interactions and confer stability to the complex. According to the results of the molecular docking studies, which are given in Figure 5, the protein-ligand interactions for the synthesized complexes were between the active residues in the 2OH4 protein and the H and Cl atoms of the ligands. It was observed that the synthesized complexes interacted with a few residue of the 2OH4 protein. While the [PdCl_2_L^1^_2_] complex interacted with the PRO-1105, HIS-1142, ARG-1122, ALA1125, MET-1123 residues, the [PdCl_2_L^2^_2_] complex interacted with the LYS-824, LEU-900, LEU-899, HIS-892, ASN-898 residues. In a similar way, the [PdCl_2_L^3^_2_] complex interacted with the GLU-1036, PHE-916, LYS-918, ARG-861, THR-862 residues and the [PdCl_2_L^4^_2_] complex interacted with the GLY-1061, ALA-1063, SER-923, LEU-1065, SER-1102 residues.

## 4. Conclusion

Currently, drug resistance is developing in cancer treatment and side effects of chemotherapy are frequently observed. Therefore, synthesis of new and effective chemotherapy agents has profound importance. In this study, novel bis(4-(4-nitrobenzyl)pyridine)dichloropalladium(II), [PdCl_2_L^1^_2_], bis(2-amino-5-bromopyridine)dichloropalladium(II), [PdCl_2_L^2^_2_], bis(2,4-dimethylpyridine)dichloropalladium(II), [PdCl_2_L^3^_2_], bis(3,4-dimethylpyridine)dichloropalladium(II), [PdCl_2_L^4^_2_] complexes were prepared. The spectroscopic techniques were used to characterization studies. In addition to experimental studies, theoretical calculations (molecular structures, potential energy distribution analysis, MEP, HOMO-LUMO orbitals and Mulliken charge distribution) were also made. Experimental results were supported by theoretical calculations. The study on the antiproliferative effects of these complexes on hepatocellular carcinoma cells (HepG2) was carried out. In our study, it was observed that the synthesized complexes had a different antiproliferative effect. According to the MTT results, the [PdCl_2_L^4^_2_] and [PdCl_2_L^2^_2_] complexes were found to be very effective even at low concentrations. The data obtained in the cell injury study were compatible with these results. This indicated that palladium complexes with pyridine ligands have antiproliferative activities on HepG2 cells. The cell based studies conducted indicated that the synthesized novel palladium complexes had cytotoxic effects on HepG2 cell line and the [PdCl_2_L^4^_2_] complex was found to be the most effective among all other complexes studied.

The interaction mechanisms between the complexes and the liver cancer protein were investigated via molecular docking. The study on the antiproliferative effects of these complexes on hepatocellular carcinoma cells (HepG2) showed that they are potent candidates for use against this liver cancer cell line. In vivo studies would allow better understanding of the metabolic effects of these compounds.

**Table S1:** Detailed assignments of experimental and theoretical wavenumbers (cm^−1^) of the complexes along with potential energy distribution (PED).

*[PdCl* *_2_* *L* *^1^* *_2_* *]*			*[PdCl* *_2_* *L* *^2^* *_2_* *]*			*[PdCl* *_2_* *L* *^3^* *_2_* *]*			*PdCl* *_2_* *L* *^4^* *_2_*		
B3LYP\LanL2dz	Scaled	Assignment	B3LYP\LanL2dz	Scaled	Assignment	B3LYP\LanL2dz	Scaled	Assignment	B3LYP\LanL2dz	Scaled	Assignment
3646	3500	ν_(OH)_99	3719	3570	ν_(NH)_95	3503	3363	ν(CH)84	3272	3141	ν(CH)94
3643	3497	ν_(OH)_84	3713	3564	ν_(NH)_83	3400	3264	ν(CH)94	3260	3130	ν(CH)96
3272	3141	ν_(CH)_91	3517	3376	ν_(NH)_84	3396	3260	ν(CH)90	3255	3125	ν(CH)94
3262	3132	ν_(CH)_92	3497	3357	ν_(NH)_92	3395	3259	ν(CH)95	3241	3111	ν(CH)99
3260	3130	ν_(CH)_97	3266	3135	ν_(CH)_90	3394	3258	ν(CH)96	3225	3096	ν(CH)95
3257	3127	ν_(CH)_97	3258	3128	ν_(CH)_89	3391	3255	ν(CH)80	3220	3091	ν(CH)90
3254	3124	ν_(CH)_89	3244	3114	ν_(CH)_93	3301	3169	ν(CH)94	3152	3026	ν(CH)80
3233	3104	ν_(CH)_89	3223	3094	ν_(CH)_94	3277	3146	ν(CH)92	3145	3019	ν(CH)79
3220	3091	ν_(CH)_95	3221	3092	ν_(CH)_94	3272	3141	ν(CH)92	3111	2987	ν(CH)99
3216	3087	ν_(CH)_91	1696	1696	δ_(HNH)_74	3117	2992	ν(CH)89	3108	2984	ν(CH)88
3197	3069	ν_(CH)_90	1688	1688	δ_(HNH)_77	3115	2990	ν(CH)95	3041	2919	ν(CH)99
3191	3063	ν_(CH)_90	1650	1650	ν_(CC)_24+δ_(HNH)_10	3093	2969	ν(CH)96	3040	2918	ν(CH)99
3087	2964	ν_(CH)_98	1648	1648	ν_(CC)_35	3089	2965	ν(CH)89	1654	1654	ν_(CC)_57
3042	2920	ν_(CH)_98	1590	1590	ν_(CC)_24+ν_(NC)_19	1682	1682	ν_(CC)_42+δ_(HCC)_10	1601	1601	ν_(CC)_19+ν_(NC)_26+δ_(CCC)_10+δ_(CCN)_21
1662	1662	ν_(CC)_57+δ_(HCC)_36	1589	1589	ν_(CC)_32+ν_(NC)_19	1631	1631	ν_(CC)_34+ ν_(NC)_12+δ_(CCC)_12+δ_(CCN)_11	1529	1529	δ_(HCC)_36+δ_(HCH)_12
1654	1654	ν_(CC)_59	1535	1535	ν_(NC)_22+δ_(HCC)_40	1628	1628	ν_(CC)_12+ ν_(NC)_12+δ_(CCC)_12	1520	1520	δ_(HCH)_64
1630	1630	ν_(CC)_63 +δ_(CCC)_10	1530	1530	ν_(NC)_22+δ_(HCC)_12	1600	1600	δ_(HCH)_58	1511	1511	δ_(HCH)_58
1593	1593	ν_(NC)_54+δ_(CCC)_10	1433	1433	δ_(HCC)_19+ν_(NC)_19	1513	1513	δ_(HCH)_50+τ_(HCCN)_12	1508	1508	δ_(HCH)_78
1539	1539	δ_(HCC)_52+ν_(CC)_15+δ_(CCC)_14+	1429	1429	δ_(HCC)_14+ν_(NC)_21+ν_(CC)_16	1499	1499	ν_(CC)_11+δ_(HCC)_26	1460	1460	δ_(HCH)_69+δ_(HCC)_10
1527	1527	δ_(HCC)_53	1372	1345	δ_(HNC)_15+ν_(NC)_11+δ_(CNC)_10	1497	1497	ν_(CC)_12+δ_(HCC)_30	1452	1452	ν_(CC)_28+δ_(HCC)_29
1508	1508	δ_(HNH)_84	1371	1344	ν_(CC)_31+δ_(HNC)_14+δ_(CCC)_11	1488	1488	δ_(HCH)_15	1448	1448	δ_(HCH)_60
1460	1460	δ_(HCC)_61 +ν_(CC)_45	1358	1331	δ_(HCC)_78		1484	δ_(HCH)_34	1357	1330	ν_(CC)_17+δ_(HCC)_55
1395	1367		1350	1323	δ_(HCC)_71		1478	δ_(HCH)_61+τ_(HCCC)_13	1321	1295	ν_(CC)_17+ν_(NC)_38
1384	1356	δ_(HCC)_17+ ν_(CC)_53	1324	1298	ν_(NC)_68	1484	1471	δ_(HCH)_50	1318	1292	ν_(CC)_19+ν_(NC)_37
1377	1349	δ_(HON)_78	1318	1292	ν_(NC)_61+δ_(HCC)_15	1478	1460	δ_(HCH)_50	1282	1256	ν_(CC)_48+δ_(HCC)_14
1359	1332	δ_(HCC)_63	1191	1167	δ_(HCC)_55	1471	1451	δ_(HCH)_26+ ν_(CC)_13	1236	1211	ν_(CC)_14+ν_(NC)_18+ δ_(HCC)_33
1306	1280	ν_(NC)_68	1190	1166	δ_(HCC)_57+ ν_(CC)_10	1460	1450	δ_(HCH)_18	1200	1176	ν_(CC)_10+ν_(NC)_10+δ_(CCC)_10+δ_(HCC)_21+ δ_(CCN)_12
1296	1270	δ_(HON)_90	1115	1093	δ_(HCC)_14+ ν_(CC)_54	1451	1367	δ_(HCH)_91	1107	1085	ν_(CC)_17+ν_(NC)_40+δ_(HCC)_14
1262	1237	ν_(CC)_12+δ_(HCC)_36	1110	1088	ν_(CC)_37	1450	1338	δ_(HCH)_90	1104	1082	ν_(CC)_16+ν_(NC)_43+δ_(HCC)_15
1240	1215	ν_(CC)_13+ ν_(NC)_12+δ_(HCC)_43	1082	1060	δ_(HNC)_53+ ν_(NC)_15	1395	1328	δ_(HCH)_19+δ_(HCC)_29	1095	1073	τ_(HCCC)_70
1229	1204	ν_(CC)_55	1078	1056	δ_(HNC)_50+ν_(NC)_13	1365	1310	δ_(HCH)_17+δ_(HCC)_32	1072	1051	τ_(HCCC)_46
1221	1197	δ_(HCC)_46+ τ_(HCCC)_12	1045	1024	δ_(CCC)_33+δ_(CCN)_13+δ_(CNC)_11	1355	1249	ν_(CC)_18+ν_(NC)_10+δ_(CCC)_13+δ_(HCC)_11	1039	1018	τ_(HCCC)_36
1206	1182	ν_(CC)_14+ ν_(NC)_24+δ_(HCC)_36	1040	1019	δ_(CCC)_34+δ_(CCN)_12+δ_(CNC)_10	1337	1247	ν_(CC)_17+δ_(CCC)_13	1020	1000	τ_(HCCC)_50
1155	1132	ν_(CC)_21+δ_(HCC)_57	1007	987	τ_(HCCC)_74	1274	1212	ν_(CC)_25+ ν_(NC)_19+δ_(HCC)_21	1005	985	τ_(HCCC)_67+τ_(CCCN)_19
1134	1111	ν_(CC)_29+δ_(HCC)_25	1006	986	τ_(HCCC)_66+ τ_(CCCC)_14	1272	1210	ν_(CC)_24+ ν_(NC)_19+δ_(HCC)_14	948	929	τ_(HCCC)_74
1088	1066	δ_(HCC)_29+δ_(CCN)_61	948	929	τ_(HCCC)_78	1237	1166	ν_(CC)_15+ ν_(NC)_11+δ_(HCC)_14	945	926	τ_(HCCC)_58+τ_(CCNC)_12
1048	1027	ν_(NC)_46+ δ_(CCN)_11	924	906	τ_(HCCC)_75	1235	1163	ν_(NC)_10+ δ_(HCC)_22	889	871	ν_(CC)_32+δ_(CCN)_21
1038	1017	δ_(HCC)_15+ δ_(CCC)_69	857	840	ν_(NC)_17+δ_(CCC)_31	1190	1110	ν_(NC)_38+ δ_(CCN)_16	869	852	τ_(HCCC)_83
1020	1000	τ_(HCCC)_66+ τ_(CCNPd)_15	855	838	δ_(CCC)_44+ ν_(NC)_18	1187	1108	ν_(NC)_37+ δ_(CCN)_17	765	750	δ_(CCN)_33+δ_(CCC)_13+δ_(CNC)_23
1017	997	τ_(HCCC)_55+ τ_(CNCC)_13	850	833	τ_(HCCC)_40+γ_(CPdCN)_12	1133	1059	τ_(HCCN)_31+ τ_(CNCC)_10	750	735	τ_(CCNC)_19+ τ_(CCCN)_16
1013	993	τ_(HCCC)_47+ τ_(CCCN)_20	848	831	τ_(HCCC)_67+γ_(CPdCN)_18	1131	1040	τ_(HCCC)_52	749	734	τ_(HCCC)_20+ τ_(CCCN)_16+τ_(CCCC)_10
1004	984	τ_(HCCN)_70	765	750	τ_(HCCC)_13+γ_(CPdCN)_15	1081	1032	τ_(HCCC)_43	572	561	γ_(CCCC)_26+γ_(CPdCN)_10
923	905	ν_(ON)_59+ ν_(OC)_10	759	744	τ_(HCCC)_12+γ_(CPdCN)_34	1061	1026	ν_(CC)_11+ν_(NC)_28+δ_(CCN)_22+δ_(CNC)_12	570	559	γ_(CCCC)_13+γ_(CPdCN)_18
906	888	τ_(HCCC)_91	689	675	ν_(PdN)_12	1053	1020	ν_(NC)_28+δ_(CCN)_21+δ_(CNC)_11	535	524	ν_(CC)_14+δ_(CCN)_22+δ_(CNC)_12+δ_(CCC)_14
895	877	τ_(HCCC)_50+	670	657	τ(_HNCN)_62	1047	1012	τ_(HCCC)_31+γ_(CCCN)_13	450	441	τ_(CCCN)_23+ γ_(CCCC)_36
888	870	ν_(ON)_46 +τ_(HCCC)_12+τ_(HCCN)_23	644	631	ν_(NC)_13+δ_(CNC)_17+τ_(HNCN)_10+ν_(BrC)_11	1041	949	τ_(HCCC)_52	447	438	τ_(CCCN)_24+ γ_(CCCC)_45
854	837	τ_(HCCC)_74	635	622	ν_(NC)_19+δ_(CNC)_14+τ_(HNCN)_16	1033	926	τ_(HCCN)_26+τ_(HCCC)_10	433	424	δ_(CCC)_75+δ_(PdNC)_10
815	799	τ_(HCCC)_10+ τ_(HCCN)_15+τ_(CCCC)_10	591	579	τ_(HNCN)_67	968	893	ν_(CC)_25+δ_(CNC)_17	430	421	δ_(CCC)_61+δ_(CNPd)_10
761	746	τ_(CCCC)_12	541	530	τ_(CCCC)_15+γ_(CPdCN)_15	945	889	ν_(CC)_17+δ_(CNC)_21		287	ν_(CC)_25+δ_(CNC)_17
746	731	τ_(CNCC)_65+ τ_(CCCC)_23	530	519	τ_(HCCC)_18+τ_(CCCC)_15	911	881	τ_(HCCC)_60	338	285	ν_(PdCl)_81
706	692	ν_(NC)_16+ ν_(ON)_21+δ_(CCC)_12	479	469	δ_(CNC)_10+ τ_(CNCC)_12+γ_(CPdCN)_12	907	744	τ_(CCCN)_29+γ_(PdCCN)_10	335	257	ν_(PdCl)_80
680	666	δ_(CCN)_66	468	459	δ_(CNC)_29+τ_(CNCC)_11	899	732	δ_(CCN)_20+ν_(NC)_11+ν_(CC)_10	302	253	δ_(NPdN)_10 +γ_(CCCC)_26
655	642	δ_(CCN)_73	460	451	τ_(HNCN)_35	759	730	δ_(CCN)_24+ν_(NC)_13+ν_(CC)_11+δ_(CCN)_10	298	3141	δ_(CCC)_64+ γ_(CCCC)_11
598	586	γ_(OCON)_53	447	438	τ_(CNCC)_16+τ_(HNCN)_19	747	558	ν_(CC)_20+ν_(PdN)_15δ_(CCC)_27			
514	504	γ_(CCCC)_50	444	435	τ_(HNCN)_39+γ_(CPdCN)_14	745	556	ν_(CC)_17+ν_(PdN)_15δ_(CCC)_25			
490	480	δ_(ONO)_34+ γ_(NCCC)_10+γ_(CCCC)_13+ γ_(OCON)_27	427	418	γ_(CPdCN)_60	569	530	τ_(CCCN)_13+γ_(CCCC)_13			
441	432	τ_(HONC)_67	337	286	ν_(PdCl)_70	567	527	ν_(CC)_11+δ_(CCN)_10+γ_(CNCC)_11+γ_(CPdCN)_12			
424	416	τ_(CCCC)_74+ τ_(HCCH)_12	334	284	ν_(PdCl)_73	541	515	τ_(CCCN)_84			
411	403	τ_(CCNPd)_53+ τ_(CNCC)_10+τ_(HCCC)_14	309	263	τ_(CCCC)_13+γ_(CPdCN)_16+γ_(BrCCC)_32	538	490	γ_(CPdCN)_12			
408	400	τ_(CNCC)_55+ τ_(HCCC)_16	302	257	τ_(CCCC)_12+γ_(CPdCN)_15+γ_(BrCCC)_35+ν_(PdCl)_11	525	426	δ_(CCC)_20+γ_(CCNPd)_13+τ_(CCCN)_15			
350	343	ν_(PdCl)_22+ δ_(CCC)_12	299	254	ν_(PdCl)_13+ ν_(BrC)_39	500	415	ν_(PdCl)_95			
339	332	ν_(PdCl)_31	294	250	ν_(BrC)_52	435	409	ν_(PdCl)_91			
312	306	ν_(PdCl)_18	262	223	ν_(BrC)_10+δ_(BrCC)_35	423	249	δ_(NPdN)_21+γ_(PdCCN)_11+γ_(CCCC)_10+γ_(CNCC)_10			
292	286	γ_(CCPdN)_12	250	213	δ_(BrCC)_48	417	228	δ_(ClPdN)_19			
261	256	δ_(CNPd)_11+ γ_(NCCC)_11+γ_(CCPdN)_14									

**Table S2:** Optimized parameters for the complexes (bond length and bond angles)

*PdCl* *_2_* *L* *^4^* *_2_*				*[PdCl* *_2_* *L* *^3^* *_2_* *]*				*[PdCl* *_2_* *L* *^2^* *_2_* *]*				*[PdCl* *_2_* *L* *^1^* *_2_* *]*			
Atoms	bond length (Å)	Atoms	bond angles (°)	Atoms	bond length (Å)	Atoms	bond angles (°)	Atoms	bond length (Å)	Atoms	bond angles (°)	Atoms	bond length (Å)	Atoms	bond angles (°)
Pd1-Cl2	2.27	Cl2-Pd1-Cl3	90.00	Pd1-Cl2	2.27	Cl2-Pd1-Cl3	90	Pd1-Cl2	2.27	Cl2-Pd1-Cl3	90	Pd1-Cl2	2.27	Cl2-Pd1-Cl3	90.00
Pd1-Cl3	2.27	Cl2-Pd1-N20	90.00	Pd1-Cl3	2.27	Cl2-Pd1-N20	90	Pd1-Cl3	2.27	Cl2-Pd1-N20	90	Pd1-Cl3	2.27	Cl2-Pd1-N22	90.00
Pd1-N20	2.05	Cl3.Pd1-N21	90.00	Pd1-N20	2.05	Cl3-Pd1-N21	90	Pd1-N20	2.05	Cl3-Pd1-N21	90	Pd1-N22	2.05	Cl3-Pd1-N23	90.00
Pd1-N21	2.05	N20-Pd1-N21	90.00	Pd1-N21	2.05	N20-Pd1-N21	90	Pd1-N21	2.05	N20-Pd1-N21	90	Pd1-N23	2.05	N22-Pd1-N23	90.00
C4-C6	1.40	C6-C4-H7	120.01	C4-C6	1.39	C6-C4-H7	120.01	C4-C6	1.39	C6-C4-H7	120.01	C4-C6	1.39	C6-C4-H7	120.01
C4-H7	1.10	C6-C4-N20	120.01	C4-H7	1.09	C6-C4-N20	120.00	C4-H7	1.09	C6-C4-N20	120.00	C4-H7	1.09	C6-C4-N22	120.01
C4-N20	1.39	H7-C4-N20	119.98	C4-N20	1.39	H7-C4-N20	119.98	C4-N20	1.39	H7-C4-N20	119.98	C4-N22	1.39	H7-C4-N22	119.98
C5-C8	1.39	C8-C5-H9	119.99	C5-C8	1.39	C8-C5-N20	120.0	C5-C8	1.39	C8-C5-N20	120.0	C5-C8	1.39	C8-C5-H9	119.99
C5-H9	1.10	C8-C5-N20	120.00	C5-C22	1.54	C8-C5-C22	119.9	C5-C22	1.47	C8-C5-C22	119.99	C5-H9	1.09	C8-C5-N22	120.00
C5-N20	1.39	H9-C5-N20	120.01	C5-N20	1.39	N20-C5-C22	120.00	C5-N20	1.39	N20-C5-C22	120.00	C5-N22	1.39	H9-C5-N22	120.01
C6-C10	1.39	C4-C6.C10	119.99	C6-C9	1.39	C4-C6-C9	119.99	C6-C9	1.39	C4-C6-C9	119.99	C6-C10	1.39	C4-C6-C10	119.99
C6-H11	1.09	C4-C6-H11	120.01	C6-H10	1.09	C4-C6-H10	120.01	C6-Br28	1.91	C4-C6-Br28	120.01	C6-H11	1.09	C4-C6-H11	120.01
C8-C10	1.39	C10.C6-H11	119.99	C8-C9	1.39	C9-C6-H10	119.99	C8-C9	1.39	C9-C6-Br28	119.99	C8-C10	1.39	C10-C6-H11	119.99
C8-C22	1.54	C5-C8-C10	120.01	C8-H11	1.09	C5-C8-C9	120.00	C8-H10	1.09	C5-C8-C9	120.00	C8-H12	1.09	C5-C8-C10	120.00
C10-C26	1.54	C5-C8-C22	119.98	C9-C30	1.54	C5-C8-H11	119.98	C9-H11	1.09	C5-C8-H10	119.98	C10-C24	1.54	C5-C8-H12	119.98
C12-C14	1.39	C10-C8-C22	120.01	C12-C14	1.39	C9-C8-H11	120.01	C12-C14	1.39	C9-C8-H10	120.01	C13-C15	1.39	C10-C8-H12	120.01
C12-H15	1.09	C6-C10-C8	119.99	C12-N21	1.39	C6-C9-C8	119.99	C12-N21	1.39	C6-C9-C8	119.99	C13-H16	1.09	C6-C10-C8	119.99
C12-N21	1.39	C6-C10-C26	119.98	C12-C26	1.54	C6-C9-C30	119.98	C12-N25	1.47	C6-C9-H11	119.98	C13-N23	1.39	C6-C10-C24	119.98
C13-C16	1.39	C8-C10-C26	120.03	C13-C15	1.39	C8-C9-C30	120.02	C13-C15	1.39	C8-C9-H11	120.02	C14-C17	1.39	C8-C10-C24	120.02
C13-H17	1.09	C14-C12-H15	120.01	C13-H16	1.09	C14-C12-N21	120.00	C13-H16	1.09	C14-C12-N21	120.00	C14-H18	1.09	C15-C13-H16	120.01
C13-N21	1.39	C14-C12-N21	120.00	C13-N21	1.39	C14-C12-C26	120.01	C13-N21	1.39	C14-C12-N25	120.01	C14-N23	1.39	C15-C13-N23	120.01
C14-C18	1.39	H15-C12-N21	119.98	C14-C17	1.39	N21-C12-C26	119.98	C14-C17	1.39	N21-C12-N25	119.98	C15-C19	1.39	H16-C13-N23	119.98
C14-C30	1.54	C16-C13-H17	119.99	C14-H18	1.09	C15-C13-H16	119.99	C14-H18	1.09	C15-C13-H16	119.99	C15-H20	1.09	C17-C14-H18	119.99
C16-C18	1.39	C16-C13-N21	120.00	C15-C17	1.39	C15-C13-N21	120.00	C15-C17	1.39	C15-C13-N21	120.00	C17-C19	1.39	C17-C14-N23	120.00
C16-H19	1.09	H17-C13-N21	120.00	C15-H19	1.09	H16-C13-N21	120.00	C15-Br29	1.91	H16-C13-N21	120.00	C17-H21	1.09	H18-C14-N23	120.01
C18-C34	1.54	C12-C14-C18	119.99	C17-C34	1.54	C12-C14-C17	119.99	C17-H19	1.09	C12-C14-C17	119.99	C19-C27	1.54	C13-C15-C19	119.99
C22-H23	1.07	C12-C14-C30	120.01	C22-H23	1.07	C12-C14-H18	120.01	N22-H23	1.00	C12-C14-H18	120.01	C24-H25	1.07	C13-C15-H20	120.01
C22-H24	1.07	C18-C14-C30	119.99	C22-H24	1.07	C17-C14-H18	119.99	N22-H24	1.00	C17-C14-H18	119.99	C24-H26	1.07	C19-C15-H20	119.99
C22-H25	1.07	C13-C16-C18	120.05	C22-H25	1.07	C13-C15-C17	120.00	N25-H26	1.00	C13-C15-C17	120.00	C24-C30	1.54	C14-C17-C19	120.00
C26-H27	1.07	C13-C16-H19	119.98	C26-H27	1.07	C13-C15-H19	119.98	N25-H27	1.00	C13-C15-Br29	119.98	C27-H28	1.07	C14-C17-H21	119.98
C26-H28	1.07	C18-C16-H19	120.01	C26-H28	1.07	C17-C15-H19	120.01			C17-C15-Br29	120.01	C27-H29	1.07	C19-C17-H21	120.01
C26-H29	1.07	C14-C18-C16	119.99	C26-H29	1.07	C14-C17-C15	119.99			C14-C17-C15	119.99	C27-C40	1.54	C15-C19-C17	119.99
C30-H31	1.07	C14-C18-C34	119.98	C30-H31	1.07	C14-C17-C34	119.98			C14-C17-H19	119.98	C30-C31	1.39	C15-C19-C27	119.98
C30-H32	1.07	C16-C18-C34	120.02	C30-H32	1.07	C15-C17-C34	120.02			C15-C17-H19	120.02	C30-C32	1.39	C17-C19-C27	120.02
C30-H33	1.07	Pd1-N20-C4	119.99	C30-H33	1.07	Pd1-N20—C4	119.99			Pd1-N20—C4	119.99	C31-C33	1.39	Pd1-N22-C4	120.00
C34-H35	1.07	Pd1-N20-C5	120.00	C34-H35	1.07	Pd1-N20-C5	120.00			Pd1-N20-C5	120.00	C31-H34	1.09	Pd1-N22-C5	120.00
C34-H36	1.07	C4-N20-C5	119.99	C34-H36	1.07	C4-N20-C5	119.99			C4-N20-C5	119.99	C32-C35	1.39	C4-N22-C5	120.00
C34-H37	1.07	Pd1-N21-C12	119.99	C34-H37	1.07	Pd1-N21-C12	119.99			Pd1-N21-C12	119.99	C32-H36	1.09	Pd1-N23-C13	120.00
		Pd1-N21-C13	120.00			Pd1-N21-C13	120.00			Pd1-N21-C13	120.00	C33-C37	1.39	Pd1-N23-C14	120.00
		C12-N21-C13	119.99			C12-N21-C13	119.99			C12-N21-C13	119.99	C33-H38	1.09	C13-N23-C14	120.00
		C8-C22-H23	109.47			C5-C22-H23	109.47			C5-C22-H23	109.47	C35-C37	1.39	C10-C24-H25	109.47
		C8-C22-H24	109.47			C5-C22-H24	109.47			C5-C22-H24	109.47	C35-H39	1.09	C10-C24-H26	109.47
		C8-C22-H25	109.47			C5-C22-H25	109.47			H23-C22-H24	109.47	C37-N50	1.47	C10-C24-C30	109.47
		H23-C22-H24	109.47			H23-C22-H24	109.47			C12-N25-H26	109.47	C40-C41	1.39		
		H23-C22-H25	109.47			H23-C22-H25	109.47			C12-N25-H27	109.47	C40-C42	1.39	H25-C24-H26	109.47
		H24-C22-H25	109.47			H24-C22-H25	109.47			H26-N25-H27	109.47	C41-C43	1.39	H25-C24-C30	109.47
		C10-C26-H27	109.47			C12-C26-H27	109.47					C41-H44	1.09	H26-C24-C30	109.47
		C10-C26-H28	109.47			C12-C26-H28	109.47					C42-C45	1.39	C19-C27-H28	109.47
		C10-C26-H29	109.47			C12-C26-H29	109.47					C42-H46	1.09	C19-C27-H29	109.47
		H27-C26-H28	109.47			H27-C26-H28	109.47					C43-C47	1.39	C19-C27-C40	109.47
		H27-C26-H29	109.47			H27-C26-H29	109.47					C43-H48	1.09	H28-C27-H29	109.47
		H28-C26-H29	109.47			H28-C26-H29	109.47					C45-C47	1.39	H28-C27-C40	109.47
		C14-C30-H31	109.47			C9-C30-H31	109.47					C45-H49	1.09	H29-C27-C40	109.47
		C14-C30-H32	109.47			C9-C30-H32	109.47					C47-N51	1.47	C24-C30-C31	120.00
		C14-C30-H33	109.47			C9-C30-H33	109.47					N50-O52	1.36	C24-C30-C32	120.00
		H31-C30-H32	109.47			H31-C30-H32	109.47					N50-O54	1.36	C31-C30-C32	120.00
		H31-C30-H33	109.47			H31-C30-H33	109.47					N51-O56	1.36	C30-C31-C33	120.01
		H32-C30-H33	109.47			H32-C30-H33	109.47					N51-O58	1.36	C30-C31-H34	119.98
		C18-C34-H35	109.47			C17-C34-H35	109.47					O52-H53	0.96	C33-C31-H34	120.01
		C18-C34-H36	109.47			C17-C34-H36	109.47					O54-55H	0.96	C30-C32-C35	120.00
		C18-C34-H37	109.47			C17-C34-H37	109.47					O56-H57	0.96	C30-C32-H36	120.01
		H35-C34-H36	109.47			H35-C34-H36	109.47					O58-H59	0.96	C35-C32-H36	119.99
		H35-C34-H37	109.47			H35-C34-H37	109.47							C31-C33-C37	119.99
		H36-C34-H37	109.47			C36-C34-H37	109.47							C31-C33-H38	120.01
														C37-C33-H38	119.99
														C32-C35-C37	120.00
														C32-C35-H39	119.98
														C37-C35-H39	120.01
														C33-C37-C35	119.99
														C33-C37.C35	119.99
														C33-C37-N50	119.98
														C35-C37-N50	120.02
														C27-C40-C41	120.00
														C27-C40-C42	120.00
														C41-C40-C42	120.00
														C40-C41-C43	120.01
														C40-C41-H44	119.98
														C43-C41-H44	120.01
														C40-C42-C45	120.00
														C40-C42-H46	120.01
														45-C42-H46	119.99
														C41-C43-C47	119.99
														C41-C43-H48	120.01
														C47-C43-H48	119.99
														C42-C45-C47	120.00
														C42-C45-H49	119.98
														C47.C45-H49	120.01
														C43-C47-C45	119.99
														C43-C47-N51	119.98
														C45-C47-N51	120.02
														C37-N50-O52	109.47
														C37-N50-O54	109.47
														O52-N50-O54	109.47
														C47-N51-O56	109.47
														C47-N51-O58	109.47
														O56-N51-O58	109.47
														N50-O52-H53	109.50
														N50-O54-H55	109.50
														N51-O56-H57	109.50

**Table S3:** Theoretical ^1^H NMR and ^13^C-NMR results for the complexes

*[PdCl* *_2_* *L* *^1^* *_2_* *]*				*[PdCl* *_2_* *L* *^2^* *_2_* *]*				*[PdCl* *_2_* *L* *^3^* *_2_* *]*					*PdCl* *_2_* *L* *^4^* *_2_*			
ATOM	Theoretical (ppm)	ATOM	Theoretical(ppm)	ATOM	Theoretical (ppm)	ATOM	Theoretical (ppm)	ATOM	Theoretical (ppm)	ATOM		Theoretical (ppm)	ATOM	Theoretical (ppm)	ATOM	Theoretical (ppm)
10,19 C	163,00	18 H	9,48	5 C	162,00	16H	8,57	5 C	174,51		7H	9,76	5 C	159,98	17H	9,24
5C	160,64	9 H	9,28	12 C	161,88	7H	8,46	12 C	172,89	11H		9,62	13 C	157,67	9H	9,14
14 C	160,46	7 H	8,34	13 C	154,65	19,11H	7,96	13 C	162,97	19,18H		8,21	12,10,18C	157,19	7H	8,12
13 C	157,79	49,16,38, 21,39,48H	8,11	4 C	154,49	18,1H	7,07	4 C	162,41	10H		8,11	4 C	154,79	15H	8,00
4C	157,63	11,34,46H	7,84	9 C	145,26	23,2H	6,91	17 C	155,26	23H		7,99	14 C	142,01	19H	7,92
47 C	156,90	12 H	7,62	17 C	145,11	26,2H	4,98	9 C	154,85	28H		5,96	8 C	141,78	11H	7,71
37 C	156,75	44,36 H	7,56	15 C	136,43			8 C	132,47	27H		4,60	6 C	131,19	28,27,35,36, 25,24H	2,48
30 C	142,43	20 H	7,47	6 C	136,26			14 C	131,65	24H		3,36	16 C	130,85	32,23,37,29, 31H	2,27
40 C	142,29	53,59 H	6,91	8 C	115,98			15 C	129,48	29H		2,76	34,26 C	27,16	33H	1,91
42,31C	136,69	57,55 H	6,82	14 C	115,81			6 C	128,88	35,32H		2,44	22 C	25,41		
41 C	136,32	29 H	4,31	5 C	162,00			26 C	42,51	25H		1,90	30 C	25,33		
32 C	136,18	26 H	4,21	12 C	161,88			22 C	38,76	31H		1,83				
6 C	132,56	28,25 H	4,07	13 C	154,65			34 C	26,86	36H		1,72				
15 C	132,45			4 C	154,49			30 C	26,77	37H		1,64				
8 C	132,16			9 C	145,26					33H		1,37				
17 C	131,95															
35 C	124,97															
43 C	124,88															
33 C	123,63															
45 C	123,20															
24 C	50,06															
27 C	50,01															

## Figures and Tables

**Scheme:**
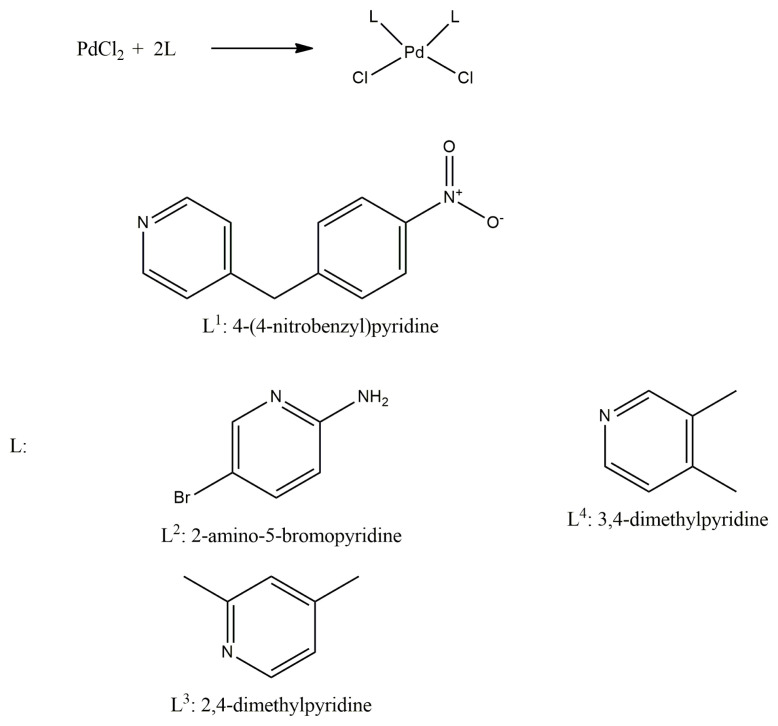
Synthesis reaction of the complexes.

**Table 1:** The selected stretching and bending vibrations of the complexes.

Complex	(N-H) cm^−1^	(Ar-H) cm^−1^	(Al-H) cm^−1^	(C=C, C=N) cm^−1^	(C-H) in-plane, cm^−1^	(C-C) in-plane, cm^−1^	(C-Br) cm^−1^	(Pd-N) cm^−1^
**[PdCl** **_2_** **L** **^1^** **_2_** **]**	-	3080	2925–2854	1614–1537	1110–1016	880–518	-	470
**[PdCl** **_2_** **L** **^2^** **_2_** **]**	3427	3076	2985–2839	1622–1402	1265–1145	823–503	889	476
**[PdCl** **_2_** **L** **^3^** **_2_** **]**	-	3030	2950–2886	1600–1473	1251–1043	885–657	-	503
**[PdCl** **_2_** **L** **^4^** **_2_** **]**	-	3035	2990–2806	1612–1415	1250–1089	856–524	-	433

**Table 2:** The ^1^H-NMR and^13^C-NMR data for the complexes.

Complexes	Ar-H ppm	Al-H ppm	N-H Ppm	Ar-C ppm	Al-C ppm
**[PdCl** **_2_** **L** **^1^** **_2_** **]**	8.64–7.42	4.24	-	152–123	40.00
**[PdCl** **_2_** **L** **^2^** **_2_** **]**	8.76–6.14	-	7.64	158–103	-
**[PdCl** **_2_** **L** **^3^** **_2_** **]**	8.40–7.65	3.31–2.26	-	150–133	17.88
**[PdCl** **_2_** **L** **^4^** **_2_** **]**	8.45–7.30	3.31–2.24	-	151–134	18.54

**Table 3:** Mulliken atomic charge of the complexes.

[PdCl_2_L^1^_2_]		[PdCl_2_L^2^_2_]		[PdCl_2_L^3^_2_]		[PdCl_2_L^4^_2_]	
Atom	Mulliken atomic charge	Atom	Mulliken atomic charge	Atom	Mulliken atomic charge	Atom	Mulliken atomic charge
1Pd	−0.053	1Pd	0.061	1Pd	−0.094	1Pd	−0.055
2Cl	−0.244	2Cl	−0.245	2Cl	−0.244	2Cl	−0.247
3Cl	−0.244	3Cl	−0.246	3Cl	−0.241	3Cl	−0.247
4C	−0.101	4C	−0.095	4C	−0.134	4C	−0.101
5C	−0.113	5C	0.238	5C	0.351	5C	−0.207
6C	−0.318	6C	−0.181	6C	−0.336	6C	−0.330
7H	0.245	7H	0.271	7H	0.244	7H	0.247
8C	−0.303	8C	−0.261	8C	−0.427	8C	0.253
9H	0.259	9C	−0.056	9C	0.419	9H	0.264
10C	0.478	10H	0.247	10H	0.233	10C	0.289
11H	0.227	11H	0.253	11H	0.241	11H	0.234
12H	0.226	12C	0.235	12C	0.380	12C	−0.231
13C	−0.106	13C	−0.100	13C	−0.139	13C	−0.107
14C	−0.112	14C	−0.262	14C	−0.423	14C	0.255
15C	−0.307	15C	−0.179	15C	−0.327	15H	0.257
16H	0.252	16H	0.280	16H	0.253	16C	−0.330
17C	−0.312	17C	−0.054	17C	0.416	17H	0.257
18H	0.254	18H	0.245	18H	0.240	18C	0.304
19C	0.478	19H	0.253	19H	0.234	19H	0.235
20H	0.227	20N	−0.256	20N	−0.286	20N	−0.253
21H	0.227	21N	−0.255	21N	−0.290	21N	−0.252
22N	−0.260	22N	−0.532	22C	−0.690	22C	−0.767
23N	−0.260	23H	0.304	23H	0.202	23H	0.245
24C	−0.673	24H	0.264	24H	0.292	24H	0.241
25H	0.213	25N	−0.550	25H	0.211	25H	0.230
26H	0.209	26H	0.266	26C	−0.731	26C	−0.757
27C	−0.673	27H	0.313	27H	0.299	27H	0.228
28H	0.212	28Br	0.020	28H	0.231	28H	0.236
29H	0.211	29Br	0.022	29H	0.222	29H	0.222
30C	0.520			30C	−0.735	30C	−0.749
31C	−0.367			31H	0.225	31H	0.235
32C	−0.367			32H	0.239	32H	0.227
33C	−0.361			33H	0.218	33H	0.229
34H	0.213			34C	−0.735	34C	−0.739
35C	−0.359			35H	0.241	35H	0.235
36H	0.213			36H	0.223	36H	0.226
37C	0.386			37H	0.219	37 H	0.221
38H	0.255						
39H	0.255						
40C	0.520						
41C	−0.366						
42C	−0.366						
43C	−0.360						
44H	0.213						
45C	−0.360						
46H	0.213						
47C	0.386						
48H	0.254						
49H	0.256						
50N	−0.048						
51N	−0.047						
52O	−0.428						
53H	0.346						
54O	−0.434						
55H	0.355						
56O	−0.428						
57H	0.346						
58O	−0.434						
59H	0.355						

**Table 4:** Some global reactivity properties of the complexes.

Parameters	[PdCl2L12] (eV)	[PdCl2L22] (eV)	[PdCl2L32] (eV)	PdCl2L42] (eV)
E_HOMO_	−5.83	−6.43	−5.60	−5.77
E_LUMO_	−2.08	−2.55	−1.88	−1.99
DE	3.75	3.88	3.72	3.78
Ionization potential (I = −E_HOMO_)	5.83	6.43	5.60	5.77
Electron affinity (A = −E_LUMO_ )	2.08	2.55	1.88	1.99
Electronegativity (χ = (I+A)/2)	6.05	4.49	3.74	3.88
Chemical potential (μ = −(I+A)/2)	−6.05	−4.49	−3.74	−3.88
Chemical hardness (η = (I−A)/2)	1.877	1.94	1.86	1.89
Chemical softness (s = 1/2η)	0.266	0.26	0.27	0.27
Electrophilic index (*w* = μ^2^/2 η)	9.74	5.19	3.76	3.98
Maximum load transfer parameter (ΔN_max_ = (I+A)/2(I−A))	1.05	1.16	1.01	1.03
